# Minimally invasive keyhole techniques for resection of giant intracranial tumors

**DOI:** 10.1186/s41016-022-00289-x

**Published:** 2022-08-05

**Authors:** Qing Lan, Michael E. Sughrue, Robert G. Briggs

**Affiliations:** 1 Department of Neurosurgery, 2nd Affiliated Hospital, Soochow University, Suzhou, China; 2 Center for Minimally Invasive Neurosurgery, Prince of Wales Private Hospital, Sydney, Australia; 3 Department of Neurosurgery, University of Oklahoma Health Science Center, Oklahoma City, Oklahoma USA

**Keywords:** Giant tumor, Keyhole approach, Meningioma, Pituitary adenoma, Schwannoma, Glioma

## Abstract

**Background:**

While keyhole neurosurgery is increasingly utilized in the operating room, there are few reports regarding the use of keyhole techniques to resect giant intracranial tumors. The feasibility and technique of that were discussed in this paper.

**Methods:**

We retrospectively reviewed 95 consecutive patients who were admitted to our service between February 2012 and September 2017 with a maximum intracranial tumor diameter >5 cm. Keyhole approaches were used to resect these tumors in each case, including supraorbital, subtemporal, suboccipital, retromastoid, frontal, temporal, occipital, parietal, pterional, a combined temporo-parietal keyhole approach, and an approach via the longitudinal fissure.

**Results:**

We achieved gross total resection in 68/95 cases (71.6%) and subtotal resection in 27/95 cases (28.4%). No surgical death or severe disabilities such as coma and limb dyskinesia occurred following surgery. At the time of discharge, 8 patients had complications related to impaired cranial nerve function. In addition, 2 patients developed hydrocephalus requiring ventriculo-peritoneal shunt placement, and 4 patients developed a postoperative CSF leak requiring surgical intervention.

**Conclusion:**

With meticulous design and reasonable selection, resection of giant intracranial tumors utilizing minimally invasive keyhole approaches can be done safely with satisfactory surgical outcomes.

**Supplementary Information:**

The online version contains supplementary material available at 10.1186/s41016-022-00289-x.

## Background

The first description of keyhole neurosurgery was reported in 1991 by Fukushima T [1]. Since that time, an increasing number of studies have described the use of keyhole approaches for the treatment of intracranial aneurysm clippings [2–6]. When the relevant intracranial vessels can be effectively protected during surgery, the principles of keyhole neurosurgery can be adapted for the treatment of intracranial tumors also. However, there is a paucity of literature describing the use of keyhole techniques to resect giant intracranial tumors. In this study, we report our surgical outcomes related to using keyhole techniques to resect giant intracranial lesions and discuss some cases.

## Methods

### Data collection

We performed a retrospective review of all patients undergoing keyhole neurosurgery for resection of a large intracranial lesion by the senior authors (QL and MS) between February 2012 and September 2017 at our home institutions. Clinical records, hospital charts, and imaging studies were reviewed through the last available follow-up. Medical histories, operative notes, and hospital courses were reviewed as well. Patients who were not seen at least 3 months postoperatively were noted as lost to follow-up. All procedures performed in studies involving human participants were in accordance with the ethical standards of our institutional research committee.

### Patient selection

Patients were included in this study if the maximum diameter of their underlying intracranial tumor was > 5 cm in any direction (i.e., coronal, sagittal, or axial). An exception to this rule was made for patients with tumors close to the brain surface.

### Operative technique

Keyhole selection was based on the location and extent of the underlying lesion. Supraorbital, subtemporal, medial suboccipital, paramedian suboccipital, retromastoid, temporal, occipital, parietal, pterional, longitudinal fissure, and combined temporo-parietal approaches were used. For each approach, a 4-cm straight incision was made for all patients for the keyhole approach [7]. The incision was hidden in the eyebrow or hairline when indicated. Hair within 1 cm of the incision was shaved. A 2.0 to 2.5 cm bone flap was created and removed in every case. After lowering the intracranial pressure via release of cerebrospinal fluid, the tumor was gradually exposed and removed in sections. Once tumor resection was compete, the bone flap was replaced and fixed without any intracranial drainage.

### Outcome measures

Patients underwent a full neurologic examination by the attending neurosurgeon immediately after surgery and at follow-up in clinic within 3 months of surgery. Thus, complications were noted in the immediate postoperative period as well as in a clinic. Postoperative magnetic resonance imaging (MRI) was reviewed within 3 days and 3 months of surgery. Tumor volumes were calculated using ImageJ (https://imagej.nih.gov/ij/).

## Results

### Tumor resection

In total, 95 patients met the inclusion criteria for this study (Table 1). The largest tumor measured 5cm×5cm×6cm, and the smallest tumor measured 5cm×2cm×3cm. The average tumor volume across all 95 patients was 92.4 cm^3^. Minimally invasive tumor resection involved 11 keyhole approaches (Table 2).

**Table 1:** Patient characteristics

Characteristic	***N***
Total patients	95
*Female*	49 (51.6%)
*Male*	46 (48.4%)
Mean age, years (range)	52.3 (23–83)
Presenting tumor	
*Meningioma*	54 (56.8%)
*Glioma*	25 (26.3%)
*Schwannoma*	12 (12.6%)
*Pituitary adenoma*	2 (2.1%)
*Metastasis*	1 (1.1%)
*Cholesteatoma*	1 (1.1%)
Mean tumor volume, cm^3^	92.4
Mean follow-up, months (range)	28.3 (10–72)

**Table 2:** Surgical outcomes

Characteristic	***N***
Keyhole approach utilized	
*Supraorbital (eyebrow)*	13 (13.7%)
*Subtemporal*	4 (4.2%)
*Suboccipital*	3 (3.2%)
*Retromastoid*	22 (23.2%)
*Temporal*	15 (15.8%)
*Frontal*	27 (28.4%)
*Pterional/mini-pterional*	5 (5.3%)
*Parietal*	3 (3.2%)
*Occipital*	1 (1.1%)
*Combined temporo-parietal*	1 (1.1%)
*Keyhole through the longitudinal fissure*	1 (1.1%)
Extent of resection	
*Gross total*	68 (71.6%)
*Subtotal*	27 (28.4%)

Gross total resection was achieved in 68/95 (71.6%) cases. In 27/95 (28.4%) cases, the tumors were subtotally resected, including 8 meningiomas (8/54, 14.8%), 1 pituitary tumor (1/2, 50%), 6 schwannomas (6/12, 50%), and 12 gliomas (12/25, 48%). In all cases, subtotal resection was intentionally performed to avoiding injuring critical brain structures which were inside of or adherent to the tumor, and in no cases did we note an unexpected or unintentional residual tumor which we “missed” a piece of.

Follow-up time ranged from 10 to 72 months, with an average of 28.3 months. In cases of gross total resection, no recurrence was detected on surveillance MRI during follow-up.

### Complications

As shown in Table 3, some complications happened, but no death or severe disability such as coma and limb dyskinesia occurred following surgery.

**Table 3:** Postoperative complications following keyhole surgery

Complications	***N***
Meningioma patients	54
*Facial numbness*	1(*petroclival meningioma*)/54 (1.9%)
*Facial weakness*^*a*^	2 /54 (1.9%)^a^
*Cerebrospinal fluid leak*^*b*^	4/54 (7.4%)^b^
Pituitary adenoma patients	2
*Diabetes insipidus w/o sequelae*^*a*^	1/2 (50%)^a^
*Diabetes insipidus w/ sequelae*^*a*^	1/2 (50%)^a^
Acoustic neuroma patients	7
*Unilateral hearing loss*	4/7 (57.1%)
*HB grade I facial paralysis*	2/7 (28.6%)
*HB grade II facial paralysis*	2/7 (28.6%)
*HB grade III facial paralysis*	1/7 (14.2%)
*HB grade IV facial paralysis*	2/7 (28.6%)
Schwannoma patients	5
*CN VI injury*	1/5 (20.0%)
*CN V injury*	2/5 (20.0%)
Glioma patients	25
*Expressive aphasia*	1/25 (4.0%)
*Mutism*^a^	2/25 (8.0%)^a^
*Neglect*	1/25 (4.0%)
*Hydrocephalus causing delirium*^c^	2/25 (8.0%)^c^
*Deep vein thrombosis*	1/25 (4.0%)

*Abbreviations: CN* cranial nerve, *HB* House-Brackkmann Scale

^a^ Temporary in patients, recovered during follow-up

^b^ Required re-operation to repair the underlying dural defect

^c^ Required placement of ventriculo-peritoneal shunt

The time of the keyhole craniotomy was less than 30 min, during which the bleeding was only a few milliliters. But 9 patients (9.47%) were transfused in the operative or perioperative periods, because of the abundant blood supply of the tumors, with an average of 540 ml erythrocyte suspending.

## Case illustrations

### Case 1: Giant pituitary adenoma, male patient, and 60 years old

**Fig. 1:**
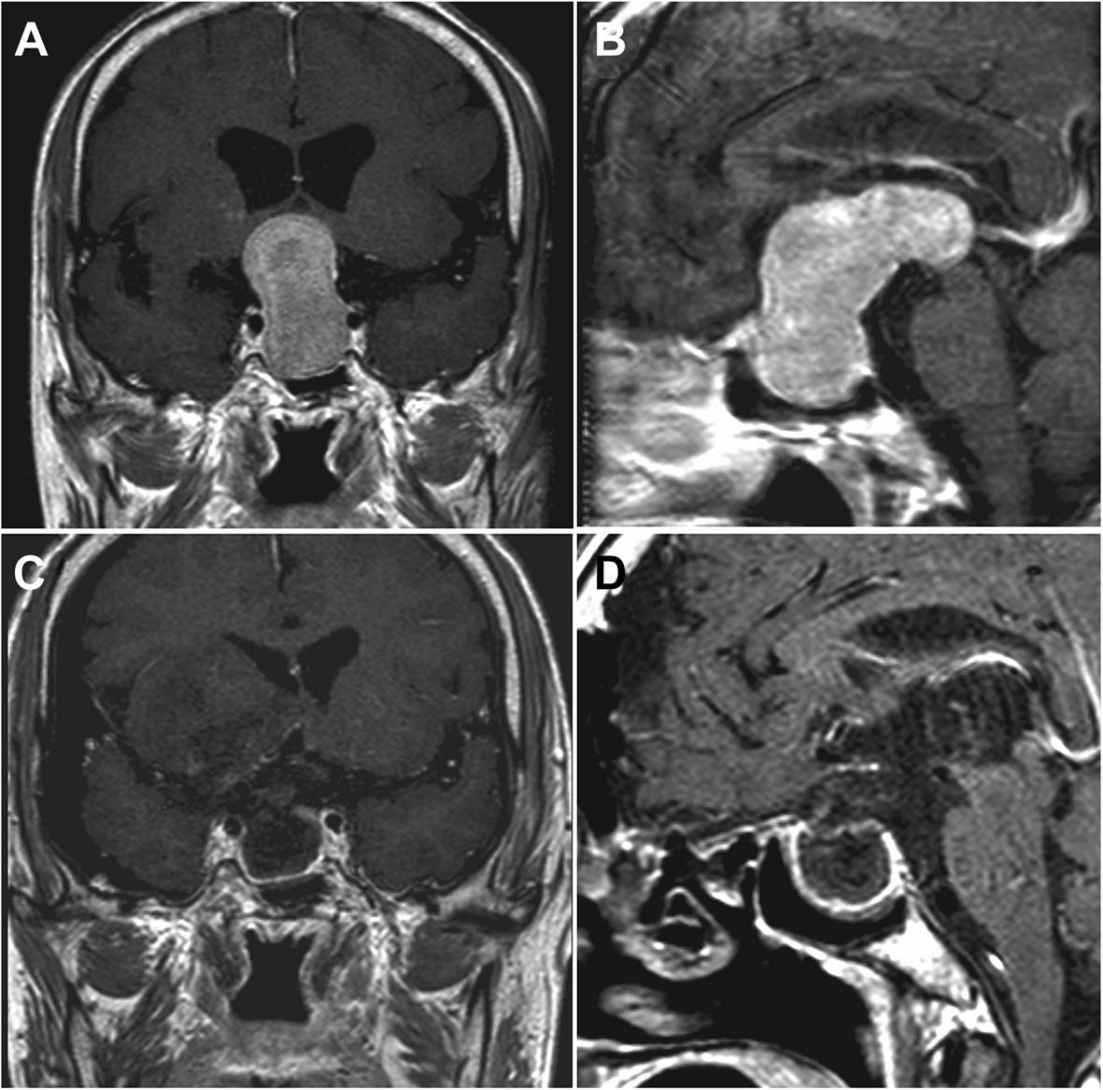
Giant pituitary adenoma resection via the supraorbital keyhole approach. **A**, **B** Pre-operation MR. **C**, **D** Postoperation MR

The sellar lesion was found 1 week after trauma. The patient had developed poor vision and bitemporal hemianopsia. PRL was 52.55 ng/mL with a low cortisol level. MRI demonstrated an irregular mass in the sellar region, measured 2.3cm×5.6cm×2.5cm. The superior aspect of the lesion extended posteriorly into the third ventricle leading to dilation of the lateral ventricles bilaterally (Fig. 1A, B).

The tumor was effectively resected via a supraorbital keyhole approach. This approach allowed for adequate visualization and excision of the posterior extent of the tumor under the microscope. During the procedure, some of the lesion was removed via curettage, and the capsule was removed by RF ablation and CUSA. The posterior portion of the tumor that had invaded the third ventricle was completely resected. In addition, the right anterior clinoid process was removed to allow for further resection of any residual tumor within the sella. The pituitary stalk was compressed to the left of the tumor and remained intact.

The patient developed increased urination and drowsiness in the first week after surgery. Following a gradual correction of elevated blood sodium levels, the patient’s mental status returned to normal. Postoperative MRI demonstrated complete tumor resection (Fig. 1C, D), and at 1 year follow-up, the patient’s thyroxine, adrenocorticotropic hormone, and PRL levels were normal. His cortisol level remained low.

### Case 2: Left frontal-temporal-occipital giant glioma, male patient, and 33 years old

Because of complaints of paroxysmal headaches, MRI was completed suggesting a possible left occipital glioma. The lesion increased gradually in size over 2 years of follow-up, and surgery was performed for partial removal. A low-grade astrocytoma was demonstrated by pathological examination.

**Fig. 2:**
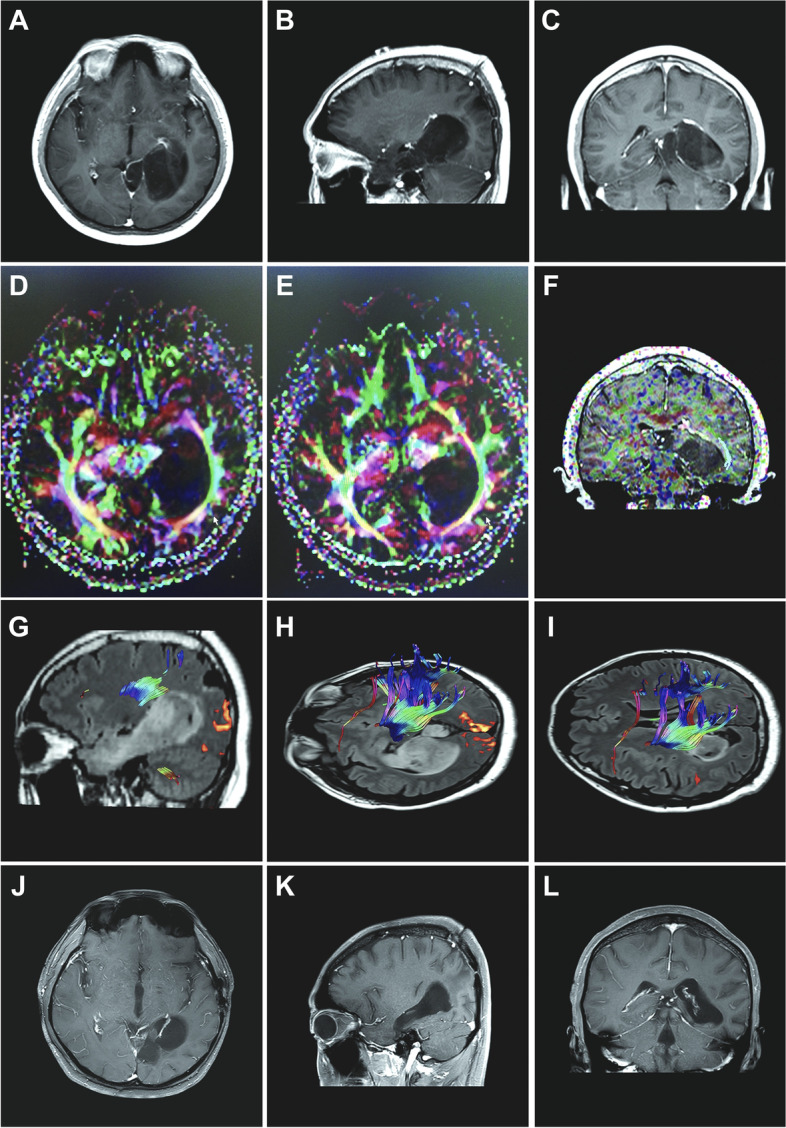
Left temporal-occipital-parietal giant glioma resection via temporoparietal combined keyhole approach. **A**–**C** Pre-operation MR images, **D**–**F** pre-operation DTI demonstrating the relative location between optic radiation and tumor, **G**–**I** pre-operation relative location between visual center, pyramidal tract, and tumor, and **J**–**L** postoperation MR images

Two years after the operation, the patient was admitted to our department for tumor progression. The patient’s physical examination revealed a U-shape flap incision in the left parietal-occipital region and a left homonymous hemianopia, suggesting that the left optic radiations were affected. MRI revealed progression of the glial tumor now located in the left deep temporal-occipital lobe with mild subtle enhancement. The lesion measured approximately 5.8cm×3.8cm× 2.9cm, with the longest axis approximately 8-cm long. The medial aspect of the tumor was compressing the brainstem and was within close proximity to the vein of Galen and the basal veins of Rosenthal. The optic radiations were displaced along the superior lateral wall of the tumor (Fig. 2A–C, D–I).

Considering that the posterior aspect of the tumor was occupying the visual center, and the lateral aspect of the tumor was occupying visual radiation and language functional areas, excision of the underlying healthy brain tissue would likely result in dysfunction. Thus, a combined keyhole approach was used to avoid these functional areas and achieve total tumor resection. The surgery was performed with a left parietal approach at first. With the guidance of the neuronavigation system, the dura was incised to create a 2.5 cm×2.0 cm flap. The tumor was subsequently accessed via the cortex channel established in the previous surgery. The tumor had a gray-white appearance and soft texture with a moderate blood supply. Pathologic examination again revealed a low-grade astrocytoma. The tumor was removed in sections, and the portion of the tumor invading the posterior fossa was resected after incising the cerebellar tentorium.

After completing the initial resection through a parietal keyhole, a left subtemporal keyhole approach was undertaken with a 2.5cm×2.5cm craniotomy. The cortex was cut near the temporal base under the optic radiations to a depth of 1.5cm to reveal the tumor. Measuring approximately 2cm×2.5cm×2.5cm, the remaining part of the tumor was removed in sections, and the passage to the parietal surgical cavity was opened. Postoperative MRI demonstrated complete tumor resection (Fig. 2J–L). After the operation, concurrent chemoradiotherapy and 5 courses of adjuvant temozolomide were performed. No tumor recurrence was observed after 5 years of follow-up.

### Case 3: Meningioma in the right atrium of lateral ventricle, male patient, and 45 years old

**Fig. 3:**
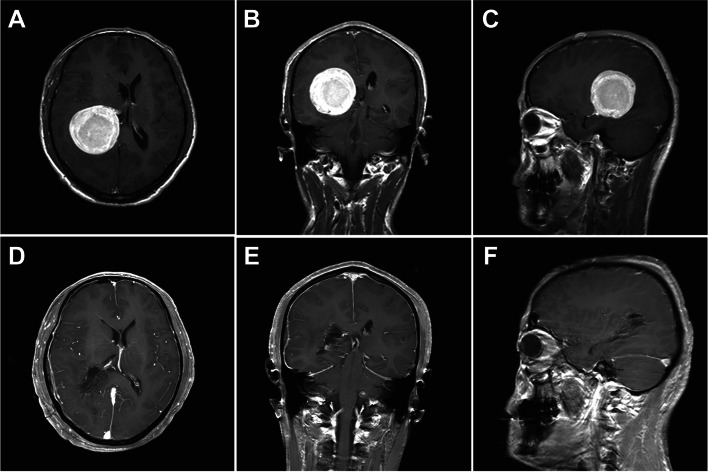
Ventricular meningioma resection via the posterior-temporal keyhole approach. **A**–**C** Pre-operation MR images. **D**–**F** postoperation MR images

This patient presented with paroxysmal headaches with a slowed mental response for nearly 1 year that had significantly worsened over the previous 2 months. MRI revealed a round lesion within the right atrium of the lateral ventricle. The lesion had a clear edge and measured 4.8cm×4.9cm×5.1cm (Fig. 3A–C).

Using a right occipital keyhole approach, an incision was made through the cortex of 1.5-cm deep to reveal the tumor. The tumor was completely covered in a capsule with a smooth surface and clear boundary. The surrounding brain tissue was edematous. The solid tumor appeared gray-red, with a rough texture and abundant blood supply. Following RF ablation through the capsule to decompress the brain, the tumor capsule was separated. The tumor was subsequently found to originate from the atrium of the lateral ventricle and was found to lie in close proximity to the choroid plexus. Postoperative imaging revealed gross total resection of the lesion (Fig. 3D–F). There were no operative complications following surgery.

### Case 4: Right trigeminal schwannoma, female patient, and 69 years old

**Fig. 4:**
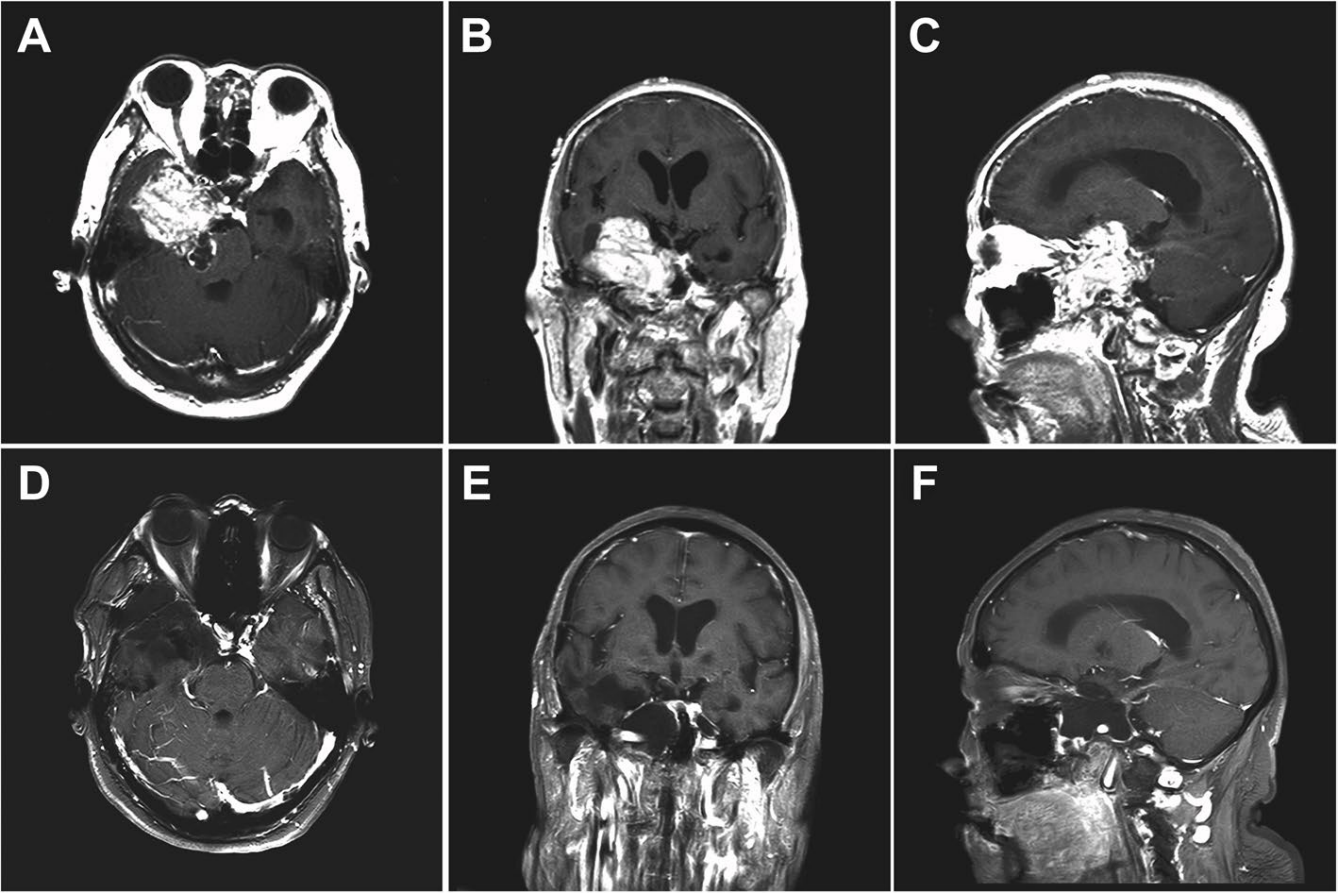
Right trigeminal schwannoma resection via right subtemporal keyhole approach. **A**–**C** Pre-operation MR images. **D**–**F** Postoperation MR images

This patient was admitted to our department due to “fatigue of the lower limbs” for half a year, but on physical examination, the patient demonstrated no obvious abnormal neurologic signs. Despite this, MRI revealed a cluster of abnormal signal changes in the right middle and posterior fossae, measuring 5.1cm ×5.3cm×5.5cm. The lesion involved the right petroclival bone and the right cavernous sinus. The cavernous segment of the internal carotid artery was obviously displaced inside the sinus. Inferiorly, the lesion involved the right ectopterygoid and protruded toward the sphenoid sinus (Fig. 4A–C).

A right subtemporal keyhole approach was undertaken, and the temporal base was lifted. At this point, it was obvious that the dura of the skull base was elevated. When cut open, a pale gray-yellow tumor with sufficient blood supply was observed and removed gross totally in sections (Fig. 4D–F) in order to reach the middle skull base. Once exposed, a thin layer of the bone could be seen having separated from the skull base sinus, the surface of the petrosal bone had been absorbed, and the full length of the internal carotid artery was exposed. The surface of the skull base seemed to be wrapped in a layer of fibrous tissue. A part of the tentorium of the cerebellum was cut, and the part of a tumor intruding into the brainstem was removed. After the operation, damage to the trochlear nerve was observed, but gradually returned to normal after discharge. No tumor recurrence was observed during 3 years of follow-up.

## Discussion

Along with technologic improvements in surgical instrumentation and medical devices, keyhole neurosurgery has been progressing rapidly with important changes in surgical concepts and methods. The indications for keyhole surgery are also gradually expanding. In this analysis, we find that keyhole approaches, when tailored to the needs of the patient, can be performed safely and efficaciously even for giant intracranial tumors based on our preliminary experience of 95 cases.

### Advantages of keyhole surgery

The goal of keyhole surgery is to simplify the approach and reduce unnecessary surgical injury. In the past, the surgical requirements for large intracranial tumors were to open the skull with a large bone flap and expose it in a wide range, so as to increase the convenience of surgical operation and strive for total resection of the tumor, even at the cost of removing some brain tissues. However, under the concept of keyhole surgery, it is completely possible to successfully remove huge tumors under a small keyhole through the accurate positioning of neuronavigation, the design of approach along the longitudinal axis of the tumor, the effective reduction of intracranial pressure, and the rapid block resection of the tumor. While reducing the surgical injury to patients, the operator can also save the operation time and energy to better deal with tumor resection.

### Total tumor resection rate

In this cohort, all tumors’ gross total resection rate was 71.6%. While the extent of resection is important, there are very few people advocating sacrificing the internal carotid or cranial nerves to push a resection of a tumor towards complete resection. In giant tumors, it is frequently unwise to attempt this, even in very experienced hands, as this is often not realistically in the patients’ best interest. The cases where we left the tumor behind were entirely due to this conscious choice and not because we could not reach the tumor in some way that removing the orbit or drilling out the entire temporal squamous bone would have made possible.

### Pituitary adenomas

The single-nostril transsphenoidal endoscopic surgery has arguably become mainstream for pituitary adenoma resection. However, for giant pituitary tumors, transcranial surgery also has its own advantages [8–10]. The adhesion between the tumor and the optic nerve and cerebral vessels can be separated outside the tumor capsule, and the lesions extending outside the internal carotid artery can be treated. The supraorbital keyhole approach with anterior to posterior viewing angle is easier to deal with backward extending tumors, such as case 1. For surgeons who are adept at endoscopic techniques, the removal of giant pituitary tumors utilizing neuroendoscopic techniques via an intracranial keyhole approach may also be appropriate.

### Gliomas

We have found that almost all gliomas can be removed through a small craniotomy, with the exception being cases where the tumor has an extensive surface component around areas which are eloquent. This is mainly due to the fact that cytoreduction of a glioma involves removing a part of the brain which is tumor involved, early in the case, which makes a substantial amount of room early in the case, allowing the visualization provided by the keyhole principle to really take over the case. When removing a giant glial tumor in the brain, designing a surgical approach along the long axis of the tumor could minimize the damage to the surface of the brain. As demonstrated in Case 2, by exposing the surface of the cortex to a depth of 2.5 cm, most of the tumor in the parietal-occipital region could be removed along the direction of long axis of the tumor while avoiding manipulation to and possible damage to critical functional areas of the cerebral cortex. Furthermore, surgery did not worsen the visual defect with which the patient presented. In fact, the patient’s homonymous hemianopia improved following surgery. When the tumor is too long to be completely removed via a single keyhole approach, a combined keyhole approach may also be an effective option. The craniotomy of a large bone flap can be simplified to two keyhole craniotomy, which avoids the influence on the cortical functional area and shortens the disadvantage of a long operation distance of a single keyhole approach. Conner AK et al. have also confirmed the feasibility of applying the principles of intracranial keyhole surgery for the removal of intrinsic gliomas [11–13].

### Intraventricular tumors

In Case 3, after using mannitol to reduce the intracranial pressure and lumbar drainage to fully release the cerebrospinal fluid, the brain tissue collapsed. A straight incision through the cortex then allowed us to access the ventricular lesion following proper retraction of the brain tissue. The blood supply of meningiomas in the ventricle is not very rich. In the process of rapid block resection of the tumor with RF ablation, there is little blood loss, and it can continuously create operation space and complete total tumor resection quickly. The method of resection and fistulation of a large brain tissue into the ventricle can be replaced by a linear incision of the cerebral cortex to better protect the brain tissue. The minimally invasive concept of a keyhole can also be reflected in the treatment of the cerebral cortex.

### Petroclival region tumors

The subtemporal keyhole approach may be viewed as a modified, minimally invasive version of the Kawase approach. Because a straight incision perpendicular to the zygomatic arch was used, the temporalis was retracted to both sides after the incision was complete. This allowed us to avoid the impediment of the zygomatic arch when flipping the temporalis flap to the skull base during the conventional craniotomy procedure. Thus, the infratemporal region can be effectively exposed without removal of the zygomatic arch. In Case 4, the petrous internal carotid artery could be clearly exposed and preserved after tumor resection. Via this approach, the tentorium cerebella could be cut open to expose the tumor underneath. If necessary, the petrous apex can also be drilled out to further expand the exposure of the petroclival region [14, 15].

### Limitations

Though these cases were performed by surgeons from multiple centers, no control group was established. But we also point out the challenge in studying this problem with a valid control group. It is extremely unlikely patients will agree to be randomized to an unnecessarily large opening versus a smaller opening if the surgeon believes they can use a smaller opening. Also, the historical comparison is flawed due to the inevitable differences in that some very big tumors cannot be successfully removed via a smaller opening. The operation time, intraoperative blood loss, postoperative hospital stay, and other postoperative neurological scores are also important for this report. But the challenge is because this group of patients include different proportions of tumors with poor blood supply or rich blood supply, tumors that are easy to remove or difficult to remove, and patients of different ages, there are great differences in the amount of bleeding, operation time, and hospital stay. For example, the amount of bleeding is 200–1200ml, the operation time is 105–695 min, and the patient’s age is 23–72 years old. Its average level is difficult to reflect and compare with the difference between routine operation. It needs to be explained more clearly by further controlled studies.

## Conclusions

This group of surgeries demonstrate that with meticulous design and reasonable selection, resection of giant intracranial tumors with single or combined keyhole approaches can be performed with at least acceptable surgical outcomes. When necessary, the posterior wall of the internal auditory canal can be opened, the anterior clinoid process and tuberculum sellae can be drilled out, and the cerebral falx and cerebellar tentorium can be cut open for full exposure and resection of the tumor. Meticulous, individualized surgical plans based on the principles of keyhole microsurgery can be applied to the resection of giant intracranial tumors. A comparative study with matched traditional craniotomy groups is worth exploring in the future.

## Supplementary Information

**Additional file 1.**

**Additional file 2.**

**Additional file 3.**

## Data Availability

The datasets generated and/or analyzed during the current study are not publicly available due individual privacy, but are available from the corresponding author on reasonable request.

## Abbreviations

RF: Radiofrequency
PRL: Prolactin
MR: Magnetic resonance
MRI: Magnetic resonance imaging
